# Unified in Our Diversity to Address Health Disparities Among Asian Americans, Native Hawaiians, and Pacific Islanders

**DOI:** 10.1089/heq.2022.0034

**Published:** 2022-07-20

**Authors:** Joseph Keawe‘aimoku Kaholokula, Mona AuYoung, Michelle Chau, Usha Sambamoorthi, Savanna Carson, Chia Thao, May Nguyen, Audrey Alo, Sheldon Riklon, Tana Lepule, Grace X. Ma

**Affiliations:** ^1^ Department of Native Hawaiian Health, John A. Burns School of Medicine, University of Hawai'i at Manoa, Honolulu, Hawai'i, USA.; ^2^ Department of General Internal Medicine and Health Services Research, University of California, Los Angeles, California, USA.; ^3^ Department of Population Health, Grossman School of Medicine, New York University, New York City, New York, USA.; ^4^ Department of Pharmacotherapy, College of Pharmacy and Texas Center for Health Disparities, University of North Texas Health Sciences Center, Fort Worth, Texas, USA.; ^5^ Department of Public Health, University of California, Merced, California, USA.; ^6^ Department of Health Systems and Population Health Sciences, College of Medicine, University of Houston College of Medicine, Houston, Texas, USA.; ^7^ Pacific Islander Health Partnership, Garden Grove, California, USA.; ^8^ Department of Family and Preventive Medicine and Center for Pacific Islander Health, University of Arkansas for Medical Sciences, Fayetteville, Arkansas, USA.; ^9^ Pacific Islander Collective of San Diego, San Diego, California, USA.; ^10^ Department of Urban Health and Population Science, Center for Asian Health and Fox Chase Cancer Center, Lewis Katz School of Medicine, Temple University, Philadelphia, Pennsylvania, USA.

**Keywords:** Asian American, Native Hawaiian, Pacific Islander, COVID-19, health disparities

## Abstract

The COVID-19 pandemic disproportionately impacted Asian Americans, Native Hawaiians, and Pacific Islanders (AA/NHPIs) in the United States. AA/NHPIs have historically been subjected to discrimination, which was exacerbated by the pandemic. To bring attention to their unique concerns, an AA/NHPI Interest Group of the National Institutes of Health Community Engagement Alliance Against COVID-19 Disparities (CEAL) was formed. This article highlights major concerns raised by the Interest Group: The pervasive and arbitrary practice of data aggregation by public health agencies and health-related researchers, the lack of culturally responsive services in the context of cultural safety, and leadership underrepresentation.

## Introduction

The coronavirus disease 2019 (COVID-19) has disproportionately impacted racial and ethnic minorities in the United States, leading to the formation of the National Institutes of Health Community Engagement Alliance (CEAL) Against COVID-19 initiative. CEAL comprises teams from 21 states across the U.S. focused on promoting diversity in vaccine and therapeutic trials to combat COVID-19 and identifying and addressing misinformation and disinformation related to COVID-19. Among the racial and ethnic minority communities of priority to CEAL are Asian Americans (AA) and Native Hawaiians and Pacific Islanders (NHPI) who have had among the highest rates of COVID-19-positive cases, hospitalizations, and deaths.^1,2^

The history of excluding AA/NHPI in national conversations, datasets, research studies, and leadership has led to devasting consequences during the COVID-19 pandemic. Because of strong advocacy by community and academic leaders, the AA/NHPI Interest Group of CEAL was formed to ensure the engagement of AA/NHPI communities. It comprises AA/NHPI community leaders and their academic partners from the various CEAL teams. This interest group came together through the advocacy efforts of researchers and community leaders within the CEAL teams. It is AA and NHPI led and represents the diversity of their communities across CEAL states. The members meet biweekly to share resources translated in multiple languages and culturally appropriate outreach strategies, as well as support one another in the work to disaggregate AA/NHPI data and advocate for policies and evidence-based practices aimed at eliminating health inequities and the disproportionate toll of COVID-19 on AA/NHPI communities.

Although AA/NHPIs represent a very diverse set of communities with unique sociocultural and socioeconomic characteristics, they share a similar history and experience of discrimination and marginalization in the U.S. that has been exacerbated by the COVID-19 pandemic with the rise in hate crimes against their communities.^3,4^ The historical mistrust toward the intentions of government and science shared by AA/NHPI communities has resurfaced during this global pandemic, in part, complicating COVID-19 mitigation efforts in these communities.^5^

To support the objectives of CEAL, the AA/NHPI Interest Group has concentrated its advocacy efforts on *two major issues* that pose significant and immediate barriers to reducing the COVID-19 burden on AA/NHPI communities across the U.S. They are the pervasive and arbitrary practice of data aggregation by public health agencies and health-related researchers and the lack of culturally responsive education and outreach strategies that account for their linguistic and cultural diversity.

## Urgent Need of Data Disaggregation on AA and NHPI

The AA and NHPI racial categories as defined by the Office of Management and Budget (OMB) Directive 15 are in and of themselves problematic because they arbitrarily aggregate very diverse racial/ethnic populations into a single race/ethnic classification.^6^ The 24 million AAs in the U.S. comprise over 20 different ethnic and national-origin groups while the 1.6 million NHPIs comprise over 12 different subgroups based on the 2020 U.S. Census. Even more concerning than the OMB racial classifications themselves is the pervasive and nonsensical practice of data aggregation of these two broad racial/ethnic groups into a single racial category or their aggregation with a mix of other racial/ethnic groups too small to separate when it comes to the analyzing and reporting of public health data. This arbitrary practice of data aggregation virtually renders the health inequities and concerns of AA/NHPI communities invisible to the government and society at large.

The COVID-19 pandemic has highlighted the importance of AA and NHPI data disaggregation. COVID-19 studies examining disaggregated AA and NHPI data have uncovered stark subgroup differences in test positivity, hospitalization, mortality, food access, and vaccine willingness and concerns.^1,7–16^ This research has flagged issues among South Asian, Chinese, Vietnamese, Filipino, Native Hawaiian, Marshallese, and other Pacific Islander communities that would otherwise have gone unnoticed. Much of this work leveraged administrative and electronic health record data due to limited research during the pandemic. However, more intentional data collection is needed to focus on the issues salient to these diverse groups. To accomplish this, it is important to engage AA/NHPI communities in the process. Working with community organizations who understand the cultures, languages, needs, and best ways to engage with and reach these subpopulations will ensure relevancy of research, diverse representation, and accuracy of data.^17,18^

As an example, a study of COVID-19 cases, hospitalizations, and deaths in Hawaii between March 2020 and February 2021 found large differences among the AA and NHPI subgroups.^19^ Among five AA subgroups, Filipino (1,247/100,000) and Vietnamese Americans (1,200/100,000) had the highest rates of COVID-19 cases with the lowest rates among Japanese (568/100,000) and Korean Americans (647/100,000), with Japanese (34/100,000) and Filipino (29/100,000) Americans having the highest death rates. Among 10 NHPI subgroups, Marshallese (10,580/100,000) and other Pacific Islanders with origins in Micronesia (8,991/100,000) had substantially higher rates of COVID-19 than all other Pacific Islander subgroups (954–7,070/100,000) and Native Hawaiians (1,181/100,000), with the death rates as high as 243/100,000 for Pacific Islanders with origins in Micronesia.

Another study in New York City found that Chinese American patients had the highest mortality rate (35.7%) of all racial and ethnic groups and South Asian patients had the highest rates of COVID-19 positivity (30.8%) and hospitalization (51.6%) among Asian patients.^9^ Had the COVID-19 data of these AA/NHPI subgroups been aggregated, these large within-group differences would have gone undocumented and unaddressed.

Despite numerous calls for data disaggregation, many national surveys continue to collect limited AA and NHPI subgroup information and present only the aggregate categories of Asian or Asian/Pacific Islander.^20–24^ Even fewer include NHPI separately.^21–25^ Datasets that do not include the collection of AA and NHPI disaggregated data, comparable to their census, should not be considered nationally representative. At a minimum, racial/ethnic data collected should follow standards set in OMB Directive 15. Ideally, we recommend collecting AA and NHPI subgroup data and disaggregating, where feasible, along with tailoring the granularity to the demographic composition or census of the county, state, or region in which the study is being conducted. These practices can increase the visibility of underrepresented groups in research and can identify potential health disparities.^26^

Efforts can also be made to pool data to create larger samples. For example, the CEAL Common Survey II encourages regional CEAL teams to collect detailed racial/ethnic subgroup data that can be aggregated for data analysis and reporting based on broader racial/ethnic categories if necessary. CEAL is an example of how community/academic partnerships can enhance research and outreach efforts.

## Cultural Safety Approach

The CEAL AA/NHPI Interest Group emphasizes the use of the *Cultural Safety* framework rather than cultural competence because it better addresses larger systemic issues, such as the need to change the inherent power differentials between academic/research organizations and communities.^27^ Cultural competence broadly focuses on a health professional's or organization's ability to interact effectively with persons from diverse cultural and socioeconomic backgrounds in ways that are static in both practice and outcome (e.g., check-the-box approach). It has been criticized for its lack of focus on the critical reflection of the inherent power dynamics that exist in society and between people and how it might play out between a health professional (e.g., implicit biases) and the persons treated or served (e.g., historical mistrust).

In contrast, cultural safety emphasizes the power differentials inherent within the larger society, the need for health professionals to reflect on their position in society and that of the person being served, and how these power dynamics can affect the care provided. Thus, cultural safety dispels the notion that acquiring some level of cultural competence is the equivalent of a deep understanding of diverse cultures, their historical context and trauma, and other lived experiences.

As a way to start shifting the power dynamics, bilingual and/or bicultural members of the AA/NHPI communities (e.g., community health workers, health care workers, community leaders) should be a crucial part of any team seeking to address health inequities in these communities. They provide valuable insight, expertise, and insider knowledge that are needed by academic-based researchers and public health organizations. For example, NHPI leaders were among the first to raise concerns about their communities being hit the hardest by COVID-19 and mobilize early on in the pandemic.^28^ They were also the first to notice that data for NHPI vaccination rates were inaccurate and misleading to local community members in some counties because of what they were experiencing on the ground.

Local AA/NHPI community health workers from the CEAL teams have been advocating for more bilingual, bicultural community health workers to help address community needs. They have also been advocating for more funding to multiple community organizations to provide COVID-19 outreach to specific marginalized communities. For example, they had to deal with the challenges that came about when a local county public health office provided disproportionately lower funding to just one organization ill equipped to meet the needs of AA/NHPI communities whose community members spoke over 25 different languages. Due to the limited number of bilingual community health workers available, community vaccination sites were not able to provide translations in all of the languages needed (e.g., Lao).

Many AA/NHPI community members with limited English proficiency had to rely on their children or other family members, some being young adults who needed to take time off from work to serve as translators so that their older family members understood the vaccination process. If we are to genuinely adopt the cultural safety approach, we need leadership and decision makers who reflect the diversity of the communities they work with, and we need health care systems set up to provide a level of care that is responsive to the cultural and linguistic needs of AA/NHPI communities.^29^

## Discussion and Recommendation

AA/NHPI communities have been disproportionally impacted by the COVID-19 pandemic, which has exacerbated the pre-existing disparities in access to health care due to structural racism and cultural barriers. The systemic lack of disaggregated data on AA/NHPI communities perpetuates the model minority myth, inequities in access to much-needed resources for the most disadvantaged communities, and maintains AA/NHPI invisibility in health disparities data and society at large. Without systematic data disaggregation in data collection, analysis, and reporting, social and health service providers and government policymakers at the local, state, and federal levels will not be able to accurately identify the needs nor allocate necessary resources to address issues to the hardest-hit AA/NHPI communities experiencing significant disparities.

To promote health equity and reduce health disparities, the CEAL AA/NHPI Interest Group has supported activities aimed to advance meaningful disaggregation of racial/ethnic data through CEAL Common Survey II, developing tip sheets of best practices to address the unique needs and disparities in AA/NHPI communities, and in-language COVID-19 resource repository. More importantly, the CEAL AA/NHPI Interest Group further confirms community observations of disproportionate challenges and impact of the COVID-19 pandemic and the critical need for policies and resources for disaggregating race/ethnicity data because aggregated data are deleterious to the health and wellbeing of AA/NHPI communities.

The CEAL AA/NHPI Interest Group has and will continue to collaborate with multilevel sectors, including community-based organizations, health centers, White House Initiative on AA/NHPI, and other government agencies to change data collection policies, implement new practices of data disaggregation, and advocate for adequate resources to provide language translation and assistance. As the first step, we must acknowledge the distinct differences between AAs and NHPIs and include them both in national data systems and research studies. Data systems and studies that do not include AA and NHPI must note and explain this substantial limitation. The ultimate goal is to further disaggregate data of AA and NHPI subgroups to observe important differences and disparities across these subgroups. This Interest Group will continue to empower CEAL teams to collect, report, and demonstrate the value of disaggregated data, unpacking the needs of the invisible and disadvantaged communities.

While there has been a push for “cultural competency” in U.S. health care settings, it is a delusion to believe that one can become culturally “competent” in any one culture besides their own. Cultural “safety” emphasizes a framework of culturally aligned safe engagement with communities and families that focuses on building trust and acknowledging power differences with the goals of improved health outcomes. The CEAL AA/NHPI Interest Group recommends CEAL teams to adopt the cultural safety approach to their community engagement, outreach, and implementation strategies when working with AA/NHPI populations during and post COVID-19 pandemic. The acknowledging and understanding historical trauma of AA/NHPI communities is needed to mitigate racial discrimination against them and provide compassionate care.^2,30–34^ Also, adequate resources are needed to support bilingual and bicultural community health workers and patient navigators who can help reduce barriers to care and mitigate susceptibility to misinformation by lending their unique experiences, the trust their communities place in them, and their cultural beliefs and practices that align with that of respective AA/NHPI communities and families.

The need for culturally and linguistically responsive services in the context of cultural safety is further supported by the diminishing returns of socioeconomic factors on health promotion observed among marginalized communities. For example, studies have found that the assumed protective effects of education and income against obesity and cigarette smoking do not always hold for AA/NHPI as they do for European Americans.^35,36^ Socioeconomic factors, such as educational attainment and income, as social determinants of health, are often the focus of understanding and addressing health inequities. However, among marginalized racial/ethnic groups, sociocultural (e.g., language barriers, acculturation stressors, immigration history), psychosocial (e.g., racism), and environmental (e.g., obesogenic neighborhoods) factors may also account for health inequities in addition to socioeconomic factors.^35,36^

Lastly, although not all AA/NHPI communities are underrepresented in health care settings, their inclusion and representation in leadership and decision-making roles have been invisible,^37^ and when there is room at the table, AA and NHPI usually just get one spot to represent all of these diverse communities. The underlying reasons for the leadership underrepresentation could be related to prejudice and discrimination, such as cultural stereotypes about the characteristics necessary to be a leader.^38,39^ The representation of AA/NHPI at leadership levels will provide an opportunity to be part of the advocacy effort for diversity, equity, and inclusion, proactively anticipating and acknowledging community needs and developing culturally linguistically appropriate solutions to enact change in reducing health disparities rather than waiting for disparities to become apparent before taking action.

## Abbreviations Used

AA: Asian Americans
CEAL: Community Engagement Alliance
COVID-19: Coronavirus disease 2019
NHPI: Native Hawaiians and Pacific Islanders
OMB: Office of Management and Budget
